# Comparison of the efficacy of aerosolized inhalation of different doses of recombinant human interferon alpha1b combined with budesonide in the treatment of asthmatic bronchitis in children

**DOI:** 10.3389/fped.2025.1642786

**Published:** 2025-10-03

**Authors:** Long Huang, Hongyu Lei, Qin Zhang, Yong Chen, Kexing Li, Delian Li

**Affiliations:** Department of Pediatrics, Longquanyi District of Chengdu Maternity & Child Health Care Hospital, Chengdu, Sichuan, China

**Keywords:** asthmatic bronchitis, bronchitis, children, recombinant human interferon alpha1b, budesonide

## Abstract

**Background:**

Asthmatic Bronchitis (AB) is the most common type of bronchitis in children, severely affecting their quality of life and growth. This study aimed to investigate the efficacy and safety of aerosol inhalation of different doses of Recombinant Human Interferon alpha1b (IFNα1b) combined with budesonide in treating childhood AB.

**Methods:**

This was a retrospective cohort study. A total of 150 children with AB treated in our hospital from December 2022 to January 2025 were retrospectively screened. They were divided into three groups based on treatment protocols: control group (budesonide alone), low-dose group (2.0 µg/kg IFNα1b+ budesonide), and high-dose group (3.0 µg/kg IFNα1b+ budesonide). The three groups were matched 1:1:1 by gender, age, disease duration, and allergy history. The primary outcomes were the remission time of cough, wheezing, and pulmonary rales. The secondary outcomes included improvements in pulmonary function indices, changes in immunoglobulin levels, and adverse events.

**Results:**

After treatment, the remission times of cough, wheezing, and pulmonary rales in the high-dose group were significantly shorter than those in the low-dose and control groups, and the low-dose group was significantly shorter than the control group (all *p* < 0.05). The high-dose group showed significantly better improvements in pulmonary function and immunoglobulin levels than the control and low-dose groups, and the low-dose group also outperformed the control group (all *p* < 0.05). There were no significant differences in adverse events among the three groups (*p* > 0.05).

**Conclusion:**

High-dose (3.0 µg/kg) IFNα1b combined with budesonide has certain advantages in improving cough, wheezing, pulmonary rales, pulmonary function, and immune status in children with AB, providing new clinical references for treatment.

## Introduction

Asthmatic bronchitis (AB) is a common acute bronchial infection in children, primarily caused by viral infections. Infections with respiratory syncytial virus, rhinovirus, influenza virus, and other pathogens can lead to respiratory tract edema, causing cellular debris to accumulate in the airways, resulting in airway obstruction and bronchial inflammation, which together manifest as AB (1, 2). Without timely intervention, the progression of AB can severely impact children's quality of life and growth development (2). Currently, the pathogenesis of AB remains unclear, though many scholars believe it is closely associated with factors such as viral infection, airway inflammation, and airway hyperresponsiveness (3, 4). Presently, the treatment of AB mainly relies on bronchodilators (5). Among them, budesonide, a glucocorticoid, exhibits potent anti-inflammatory, anti-allergic, and immunosuppressive effects, effectively relieving wheezing symptoms (6). Bian et al. (6) demonstrated that the combination of budesonide in treating childhood AB significantly outperforms traditional therapies. However, while budesonide can effectively control symptoms and inflammation, it has limitations in modifying the disease course and regulating immune function. In this study, the diagnosis of AB was made according to the 8th edition of Practical Pediatrics edited by Zhu Futang (7), which is the most authoritative textbook in Chinese pediatric practice. Under this definition, AB mainly refers to preschool children (aged 1–3 years) with recurrent wheezing, cough, and pulmonary rales, often triggered by viral infections. This entity clinically overlaps with “viral-induced wheeze” or “preschool wheeze” as described in international guidelines (GINA, ERS, ATS) (3, 8, 9), though the nomenclature differs.

Recombinant human interferon exerts antiviral and immunomodulatory effects (10). Recombinant human interferon alpha1b (IFN*α*1b) represents the predominant antiviral subtype in the Chinese population (10, 11). In the treatment of AB, combining IFN*α*1b with budesonide holds promise for enhancing therapeutic efficacy by regulating immune status, inhibiting viral replication, and reducing airway inflammation (11, 12). Bai et al. (12) confirmed the clinical efficacy and safety of aerosol inhalation of IFNα1b combined with budesonide in children with AB. Nevertheless, the optimal dosage of IFNα1b and the comparative efficacy of different dosages when combined with budesonide in treating childhood AB remain inadequately studied.

Therefore, conducting a retrospective study to systematically compare the efficacy and safety of aerosol inhalation of different doses of IFNα1b combined with budesonide in treating childhood AB is of significant clinical importance. This study aims to identify the optimal IFNα1b dosage by analyzing clinical efficacy indicators, symptom remission times, and adverse events associated with different dosage combinations. The findings seek to provide more precise data support for optimizing clinical treatment strategies.

## Materials and methods

### Patient selection

This was a retrospective cohort study. Records of 150 children with AB treated in our hospital from December 2022 to January 2025 were retrospectively selected. According to different treatment regimens, the patients were divided into three groups: the control group (budesonide monotherapy), the low-dose group (2.0 µg/kg IFNα1b combined with budesonide), and the high-dose group (3.0 µg/kg IFNα1b combined with budesonide). The three groups were matched 1:1:1 based on gender, age, disease duration, and allergy history.

#### Inclusion criteria

• Meeting the diagnostic criteria for AB (7);
• Aged between 0 and 3 years;
• Stable vital signs of the children;
• Receiving treatment with budesonide or a combination of IFN*α*1b and budesonide.

#### Exclusion criteria

• Comorbid with severe underlying diseases such as congenital cardiopulmonary diseases and immunodeficiency diseases;
• Suffering from functional disorders like bronchial foreign bodies;
• Children with impaired major organ functions or severe malnutrition;
• Administration of other immunomodulatory drugs and antiviral drugs during treatment.

The diagnosis of AB patients was made according to Practical Pediatrics (8th edition) by Zhu Futang (7). These criteria include acute onset, characterized by a short course and rapid progression, often accompanied by symptoms of upper respiratory tract infection, such as sore throat and nasal congestion. Patients frequently present with irritative dry cough or expectoration of a small amount of mucoid sputum and may experience a sensation of chest tightness. When not coughing, the sounds of sputum and wheezing can often be heard in the throat, although there is no obvious dyspnea. Cough and wheezing worsen at night or in the early morning, similar to asthma, and severe wheezing can lead to cyanosis. Complete blood count examination usually reveals abnormalities in white blood cells or neutrophils. While this definition is widely used in China, it differs from the international consensus terminology, where “asthmatic bronchitis” is rarely used. Instead, terms such as “viral-induced wheeze,” “recurrent wheeze,” or “preschool wheeze” are preferred.

### Treatment protocols

#### Control group

Children received routine treatments including expectoration, oxygen inhalation, anti-infection, and anti-asthmatic therapy. Oxygen-driven inhalation of budesonide (suspension) (specification: 2 ml: 1.0 mg) was administered twice daily, lasting 5–10 minutes each time, for 5–7 consecutive days.

#### Low-dose group

On the basis of the control group's treatment, aerosol inhalation of 2.0 µg/kg IFNα1b was added, administered twice daily for 5–7 consecutive days.

#### High-dose group

Based on the low-dose group's treatment, the dose of IFN*α*1b was increased to 3.0 µg/kg.

#### Primary outcomes

Remission time of cough, wheezing, and pulmonary rales.

#### Secondary outcomes

Pulmonary function improvement before and after treatment: Tidal volume (VT, ml/kg), time to peak expiratory flow (TPTEF)/expiratory time (TE), and respiratory rate (RR, breaths/min) were measured using a MasterScreen Paediatric Spirometry system (Jaeger, Germany), which has been validated for infants and preschool children aged 1–3 years. Measurements were performed by tidal breathing analysis, a method widely accepted for this age group. All assessments were conducted with the children awake and calm in a quiet environment. Each parameter was measured at least three times, and the best result was recorded. All tests were performed by pediatric respiratory technicians with standardized training to ensure reliability. Changes in immunoglobulin levels before and after treatment: 3 ml of venous blood was collected from each child, centrifuged (3000 rpm for 10 min), and the upper-layer serum was extracted. The levels of immunoglobulin G (IgG, g/L), immunoglobulin A (IgA, g/L), and immunoglobulin M (IgM, g/L) were detected using a Beckman Coulter IMMAGE 800 specific protein analyzer and corresponding reagents. Adverse events (defined as adverse events that started or worsened after the first drug dose) included gastrointestinal discomfort, nausea, vomiting, and allergies.

### Statistical methods

Data were entered into Microsoft Excel and analyzed using SPSS version 25.0 (IBM Corp, Armonk, NY, USA). Continuous variables were reported as mean and standard deviation (SD) or median and interquartile range (IQR) based on normality assessed by the Shapiro–Wilk test. Normally distributed data were analyzed using one-way analysis of variance (ANOVA) to evaluate statistical differences among the three groups, with *post-hoc* pairwise comparisons performed using the LSD method. Non-normally distributed data were analyzed using the Kruskal–Wallis H test for intergroup differences, and pairwise comparisons were conducted using the Nemenyi test. Categorical variables were reported as frequencies and percentages, and differences among the three groups were evaluated using the chi-square test. A *p*-value less than 0.05 was considered statistically significant. All reported *p*-values were two-sided.

## Results

**Figure 1:**
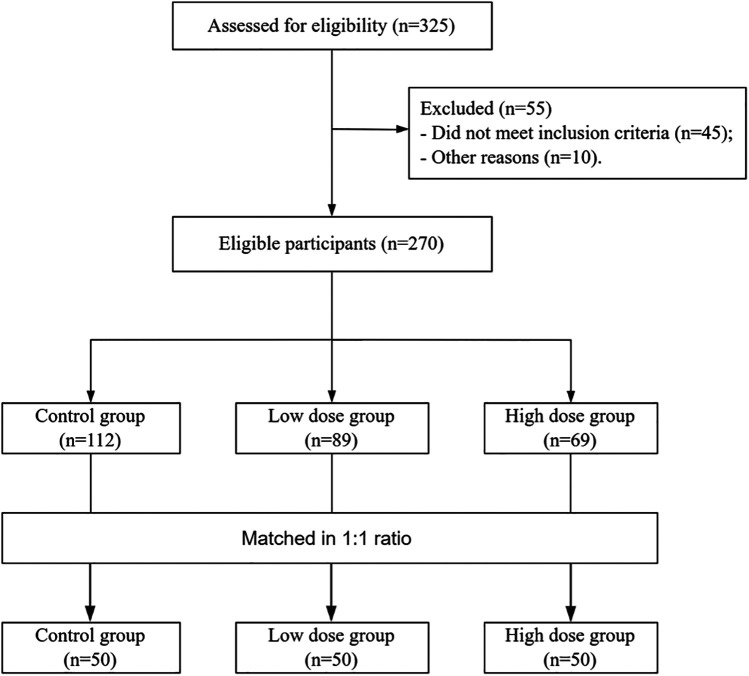
Patient screening flowchart.

A total of 150 enrolled children were matched into three groups at a 1:1:1 ratio (Figure 1). There were no statistically significant differences in baseline characteristics such as gender, age, disease duration, or allergy history among the three groups (all *p* > 0.05) (Table 1).

**Table 1:** Comparison of basic characteristics of children in three groups.

Characteristics	Control group (*n* = 50)	Low—dose group (*n* = 50)	High—dose group (*n* = 50)	*χ^2^*/H	*p*
Male, *n* (%)	28 (56.0)	23 (46.0)	31 (62.0)	2.636	0.268
Age (Years)				6.831	0.145
<1	9 (18.0)	14 (28.0)	7 (14.0)		
1–2	19 (38.0)	10 (20.0)	13 (26.0)		
>2	22 (44.0)	26 (52.0)	30 (60.0)		
Course of disease (Days)	5 (3–5)	4 (3–5)	4 (3–5)	1.009	0.604
Allergy history	30 (60.0)	24 (48.0)	26 (52.0)	1.500	0.472

Values are expressed as n (%) or median (IQR) as appropriate. IQR, interquartile range.

After treatment, the remission times of cough, wheezing, and pulmonary rales in the high—dose group were significantly shorter than those in the low—dose group and the control group, and the remission time in the low—dose group was significantly shorter than that in the control group (all *p* < 0.05) (Table 2).

**Table 2:** Comparison of clinical symptom remission of children in three groups, days.

Symptom remission	Control group (*n* = 50)	Low—dose group (*n* = 50)	High—dose group (*n* = 50)	H	*p*
Cough	9 (7–10)	7 (5–9)^a^	6 (5–7)^a^^,^^b^	20.053	<0.001
Wheezing	6 (5–8)	5 (4–7)^a^	4.5 (3–5)^a^^,^^b^	23.671	<0.001
Pulmonary rales	7 (5–8)	5 (4–7)^a^	4 (3–5)^a^^,^^b^	20.269	<0.001

Compared with the control group, ^a^*p* < 0.05; compared with the low—dose group, ^b^*p* < 0.05. Values are expressed as median (IQR). IQR, interquartile range.

Before treatment, there were no significant differences in the levels of VT, TPTEF/TE, and RR among the three groups of children (all *p* > 0.05). After treatment, the VT and TPTEF/TE in the high—dose group were significantly higher than those in the other two groups, and the low—dose group had higher levels than the control group; while the RR in the high—dose group was significantly lower than that in the two groups, and the low—dose group had a lower RR than the control group (all *p* < 0.05) (Table 3).

**Table 3:** Comparison of pulmonary function indicators among three groups.

Group	Time	Control group (*n* = 50)	Low—dose group (*n* = 50)	High—dose group (*n* = 50)	*F/H*	*p*
VT (ml/kg)	Before treatment	5.77 ± 0.95	5.64 ± 1.19	5.61 ± 1.11	0.294	0.745
	After treatment	6.25 ± 1.16	6.85 ± 1.41^a^	7.70 ± 1.80^a^^,^^b^	12.137	<0.01
TPTEF/TE (%)	Before treatment	18.38 (15.82–22.85)	19.9 (14.97–23.94)	21.41 (15.91–24.94)	3.491	0.175
	After treatment	21.51 ± 5.04	27.57 ± 6.14^a^	34.33 ± 7.18^a^^,^^b^	53.775	<0.01
RR (times/min)	Before treatment	43.04 ± 4.97	45.16 ± 6.18	43.98 ± 5.54	1.809	0.167
	After treatment	28 (25–32)	26 (25–28)^a^	24 (21–25)^a^^,^^b^	39.812	<0.01

Compared with the control group, ^a^*p* < 0.05; compared with the low—dose group, ^b^*p* < 0.05. Values are expressed as mean ± SD or median (IQR) as appropriate. VT, tidal volume; TPTEF/TE, time to peak tidal expiratory flow/expiratory time; RR, respiratory rate; SD, standard deviation; IQR, interquartile range.

Before treatment, there were no significant differences in the levels of IgG, IgA, and IgM among the three groups of children (all *p* > 0.05). After treatment, the levels of IgG, IgA, and IgM in the high—dose group were significantly higher than those in the other two groups, and the levels in the low—dose group were significantly higher than those in the control group (all *p* < 0.05) (Table 4).

**Table 4:** Comparison of immunoglobulin levels in children among three groups.

Group	Time	Control group (*n* = 50)	Low—dose group (*n* = 50)	High—dose group (*n* = 50)	*F/H*	*p*
IgG(g/L)	Before treatment	7.91 ± 0.94	7.78 ± 1.16	7.96 ± 1.08	0.381	0.684
	After treatment	9.06 ± 1.26	9.91 ± 1.44^a^	10.57 ± 1.53^a^^,^^b^	14.258	<0.01
IgA(g/L)	Before treatment	0.97 (0.88–1.15)	1.09 (0.92–1.26)	0.96 (0.77–1.24)	2.674	0.263
	After treatment	1.25 ± 0.21	1.53 ± 0.28^a^	1.70 ± 0.34^a^^,^^b^	32.603	<0.01
IgM(g/L)	Before treatment	0.94 ± 0.20	0.91 ± 0.23	0.96 ± 0.25	0.647	0.525
	After treatment	1.25 ± 0.26	1.52 ± 0.34^a^	1.77 ± 0.30^a^^,^^b^	35.965	<0.01

Compared with the control group, ^a^*p* < 0.05; compared with the low—dose group, ^b^*p* < 0.05. Values are expressed as mean ± SD or median (IQR). IgG, immunoglobulin G; IgA, immunoglobulin A; IgM, immunoglobulin M; SD, standard deviation; IQR, interquartile range.

In the control group (*n* = 50), there were 2 cases of stomach discomfort, 3 cases of nausea/vomiting, and 3 cases of allergy, totaling 8 events (16%). In the low-dose group (*n* = 50), there were 3 cases of stomach discomfort, 4 cases of nausea/vomiting, and 5 cases of allergy, totaling 11 events (22%). In the high-dose group (*n* = 50), there were 5 cases of stomach discomfort, 7 cases of nausea/vomiting, and 5 cases of allergy, totaling 17 events (34%). Importantly, all adverse events were mild to moderate in severity, resolved with symptomatic management, and no serious adverse events were observed. There were no statistically significant differences in the incidence of each individual adverse event or the total number of events among the three groups (*p* > 0.05) (Table 5).

**Table 5:** Adverse event occurrences in three groups of patients.

Adverse events	Control group (*n* = 50)	Low—dose group (*n* = 50)	High—dose group (*n* = 50)	*χ^2^*	*p*
Stomach discomfort	2 (4.0)	3 (6.0)	5 (10.0)	1.500	0.472
Nausea and vomiting	3 (6.0)	4 (8.0)	7 (14.0)	2.048	0.359
Allergy	3 (6.0)	5 (10.0)	5 (10.0)	0.674	0.714
Total number of occurrences	8 (16.0)	12 (24.0)	17 (34.0)	4.605	0.100

Values are expressed as *n* (%). Adverse events included stomach discomfort, nausea/vomiting, and allergy.

## Discussion

The results of this study showed that in the treatment of pediatric AB with high—dose IFNα1b combined with budesonide, the remission times of cough, wheezing, and pulmonary rales were significantly shorter than those in the control group and the low—dose IFNα1b combined with budesonide group. In terms of improving pulmonary function indicators and immunoglobulin levels, it was significantly better than the control group and the low—dose IFNα1b combined with budesonide group, and the low—dose combination with budesonide was better than budesonide monotherapy. Moreover, there was no significant difference in the incidence of adverse events among the three groups. This indicated that on the basis of budesonide, the combined treatment with an increased dose of IFNα1b had a more significant therapeutic effect and also had safety. Shang et al. (13) in a multi—center RCT confirmed that adding a 4.0 μg/kg dose of IFN*α*1b to the basic treatment for children with acute bronchiolitis had better symptom improvement than a 2.0 μg/kg dose. Luo et al. (14) in a meta—analysis including 13 RCTs also found that compared with low—dose IFNα1b, high—dose IFNα1b in the treatment of children with bronchiolitis could shorten the hospital stay, the disappearance time of three depression signs, and the disappearance time of wheezing. Similarly, this study confirmed that high—dose IFNα1b had more advantages in improving pulmonary function indicators and immunoglobulin levels in the treatment of pediatric AB. Our choice of 2.0 µg/kg and 3.0 µg/kg regimens was consistent with the dosing ranges reported in the literature. Previous Chinese studies demonstrated the efficacy and safety of 2.0 µg/kg (15–17), while multicenter RCTs and meta-analyses suggested that higher doses (3–4 µg/kg) could further improve clinical outcomes (14,15). By selecting 3.0 µg/kg as the high-dose regimen, we sought to align with this evidence while maintaining tolerability in the preschool age group. Mainly because budesonide can inhibit the migration and activation of a variety of inflammatory cells, reduce the release of inflammatory mediators (such as leukotrienes, histamine, prostaglandins, etc.), alleviate the congestion and edema of the airway mucosa, reduce the inflammatory response of the airway, and relieve asthma symptoms (6, 15). IFNα1b regulates immune function, reduces the airway inflammatory response, reduces the stimulation of airway smooth muscle by inflammatory mediators, and synergizes with budesonide to further improve pulmonary function (13, 14). High—dose IFN*α*1b may play a stronger role in immune regulation and anti—inflammation, thus leading to a more significant improvement in pulmonary function (11–14). However, Xu et al. (16) found in their study that on the basis of conventional treatment, the combined nebulization inhalation of low—dose (2.0 μg/kg) IFNα1b in the treatment of children with acute bronchiolitis had more advantages than 4.0 μg/kg. In addition, Huang (17) also found that low—dose (2.0 μg/kg) IFNα1b had a better therapeutic effect on children with bronchiolitis. This is different from the results of this study. This may be related to the different individual immune statuses of the children. High—dose IFN*α*1b may further enhance the immune response, trigger the excessive release of inflammatory mediators, and aggravate immune disorders, thus resulting in no obvious improvement in symptoms (16, 17). In addition, factors such as the drug formulation, nebulization device, and treatment course used during the treatment may also lead to different results. Therefore, multi—center and high—quality studies are still needed in the future to explore the optimal treatment dose.

TPTEF/TE can reflect the obstruction of small airways and is a main detection index of lung function in children with small airway obstruction. The more severe the obstruction, the more significant the reduction in TPTEF/TE (18, 19). The greater the airway resistance, the higher the RR value (19, 20). Therefore, the progression of the children's condition can be evaluated through the above indicators. The results of this study also showed that after treatment, the improvement in the levels of VT, TPTEF/TE, and RR in the high—dose group was better than that in the other two groups.

Pediatric AB is often accompanied by immune dysfunction. Changes in the levels of immunoglobulins such as IgG, IgA, and IgM reflect the immune status of the body. The results of this study showed that after treatment, the levels of IgG, IgA, and IgM in the high—dose group were significantly higher than those in the other two groups. This is consistent with the research results of Guo (21) and Ge et al. (22). This is because IFNα1b can regulate the function of the body's immune cells, promote the synthesis and secretion of immunoglobulins, and enhance the body's immune defense ability. High—dose IFNα1b may more effectively activate the immune regulation pathway, thus leading to a more obvious increase in immunoglobulin levels (13, 14, 21–23).

In terms of adverse events, a certain proportion of children in all three groups experienced stomach discomfort, nausea and vomiting, and allergies. In terms of adverse events, a certain proportion of children in all three groups experienced stomach discomfort, nausea/vomiting, and allergies. All of these events were classified as mild to moderate and resolved with symptomatic treatment, and importantly, no serious adverse events occurred in any group. There were no significant differences in the comparison of individual adverse events or the total number of cases among the three groups, suggesting that increasing the dose of IFNα1b did not significantly increase the overall risk of adverse reactions. This indicates that the treatment of different doses of IFNα1b combined with budesonide has similarity in terms of safety, and the risk of adverse events is not significantly increased due to the increase in dose. In conclusion, this study confirmed that high—dose (3.0 µg/kg) IFNα1b has certain advantages and safety in the treatment of children with AB. This helps doctors to more targetedly select treatment plans when facing children with AB, improve the treatment effect, and improve the prognosis of patients. However, because the individual conditions of children are different, how to determine the most accurate individualized treatment dose to achieve the best efficacy and the least side effects still requires further multi—center and high—quality research.

It is important to note that the terminology of “asthmatic bronchitis” (AB) used in this study is based on the diagnostic criteria of Zhu Futang's Practical Pediatrics (8th edition) (7), which is authoritative in Chinese pediatric practice. In international guidelines (GINA, ERS, ATS) (3, 8, 9), however, this entity is more commonly referred to as “viral-induced wheeze” or “preschool wheeze.” Although the nomenclature differs, the clinical features substantially overlap. By clarifying this point, we aim to minimize confusion for international readers and to highlight the consistency of our findings with the broader pediatric respiratory literature.

This study has some limitations. It is a single—center retrospective analysis with a limited sample size, which affects the universality and reliability of the research results. The effect and safety of long—term use of high—dose IFN*α*1b cannot be evaluated. The long—term side effects of some drugs may not appear in the short term, and the long—term changes in immune function also require longer—term follow—up observation. Due to the young age of the children and their limited expression ability, some adverse reactions may be underreported, which affects the statistics of the true incidence and severity of complications. In conclusion, multi—center and high—quality research needs to be carried out in the future to explore the optimal dose of IFNα1b.

## Conclusion

Nebulized inhalation of high—dose IFNα1b combined with budesonide in the treatment of pediatric AB has significant advantages in improving lung function and immunoglobulin levels, without increasing the incidence of adverse events. It provides a more effective treatment regimen for the clinical treatment of pediatric AB. However, in the future, further multi—center and high—quality studies are needed to more comprehensively explore the optimal dose and safety of IFNα1b.

## Data Availability

The original contributions presented in the study are included in the article/Supplementary Material, further inquiries can be directed to the corresponding author.
